# Where is Waldo? or find the platelet

**DOI:** 10.1007/s10555-021-09985-z

**Published:** 2021-09-30

**Authors:** D. G. Menter

**Affiliations:** Department of Gastrointestinal Medical Oncology, The University of Texas MD Anderson Cancer Center, Houston, TX USA

**Keywords:** Coagulation, Platelets, Viral infection, Cancer, Metastasis, Immune response

## Abstract

Platelets evolved from nucleated thrombocytes that exhibit both coagulation and immune function. The essential role of platelets in coagulation is common knowledge. The larger and critical role of platelets in immune responses and cancer are frequently overlooked in our modern-day, large-data-set, sequencing-oriented efforts. Much like Waldo, their small size, biophysical characteristics, rapid biological responses, active cytoskeleton, migration capacity, and lack of a nucleus make them difficult to track as single platelets disappear while executing their function into the histologic “tissue scape”. The adaptive evolution of platelets is linked to placentalization and stopping massive blood loss. This resulted in exclusion of any platelet nucleus and therefore sustainable gene expression due to being extruded in the billions (1011) per day from megakaryocytes under bone marrow protection. The platelets’ small size and sheer number in circulation, combined with an active open canalicular exchange- and membrane-reserve system, plus an array of pathogen receptors enable them to deal with small pathogenic viral treats and to decorate larger ones for further immune identification and immune-cell recruitment. Once stimulated, platelets release most serum-based cytokines and growth factors that contribute to cell growth and wound repair, and potentially to immune suppression. From a self-taught practitioner of the illustrative arts with a ken for platelet biology, this offering is a humble attempt to provide a stimulating sketch of the critical importance of platelet biology and insights into potential new directions for finding the Waldo-esque platelet.

## Where is Waldo? Tomb of the unknown platelet

In order to establish a Waldo analogy frame of reference for the complexity associated with the study of platelets, it may help to understand context for the uninitiated. Where is Wally or Waldo? is a series of children’s search-based puzzle books created by English illustrator Martin Handford. Each book contains brightly colored illustrations that detail elaborate amusing scenes with so many individual characters that it is difficult to find a single tiny Waldo image (https://waldo.fandom.com/wiki/Where%27s_Waldo%3F). This complexity is analogous to attempting to find and characterize a single tiny platelet in a histopathology sample.

**Fig. 1:**
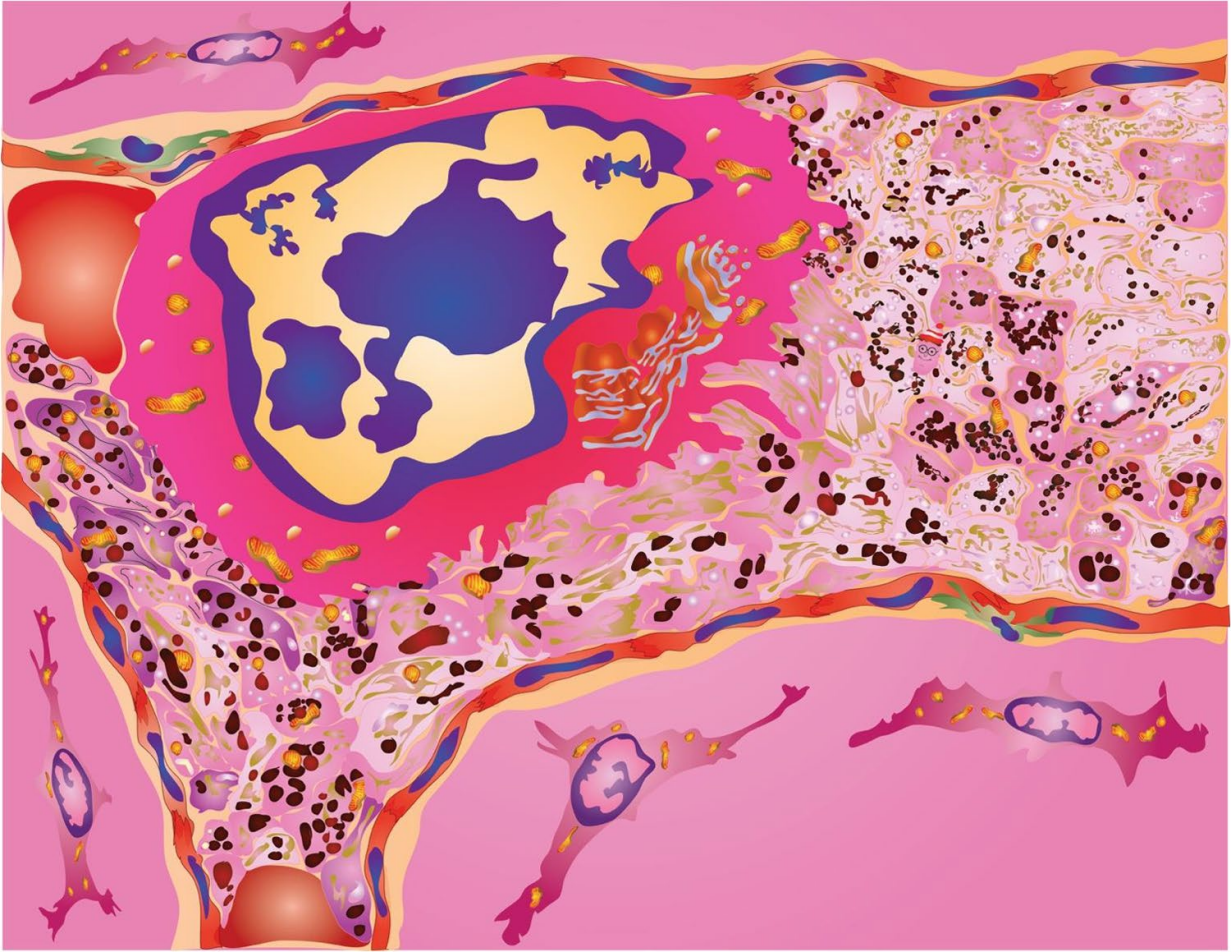
Where is Waldo? or find the platelet. Arrested tumor cell surrounded by activated platelets, other interactions, and possibly a micro-Wally

As critical anucleate elements or expendable immune and hemostatic systems, platelets essentially serve as essentially drones and, after completing their function, platelets are consumed into amorphous aggregates (Fig. 1). This lack of nucleus enables the platelet migratory behavior to rapidly merge into surrounding tissues; however, it also makes the behavior short lived due to lack of renewal capability. As a result, the anucleate platelet is often overlooked in pathological cell analyses unless specifically accounted for and confirmed by immunohistochemistry or other approach. The dramatic morphological changes in microtubules, actin polymerization, and granule release are best observed by electron microscopy. Their expendability—active cytoskeleton and discharge of any recognizable granular structures—leads to a merging into or transient entombment within the surrounding tissues.

## A passion for platelets or the fun never stops

Our Waldo-platelet analogy is complicated further by current sequencing technology that is now used now to characterize single cells. In today’s modern era of single-cell sequencing, T-cell receptor sequencing, and other forms of large data-set tissue analyses, the study of the Waldo-esque platelet remains consistently neglected due to nonoptimal DNA- and RNA- sequencing technologies. Essential for survival, this ignored cell has evolved phylogenetically over time from a nucleated-thrombocyte immune cell with dual hemostatic function into anucleate marsupial and mammal platelets. Without a nucleus, the capacity of platelets to maintain an extended biologic response in local tissue is limited by the lack of long-term-adaptation responses through new gene expression. In the case of platelets, DNA- and RNA- sequencing technologies can examine the ability of platelets to exchange circulatory information through a specialized open canalicular system (OCS) of membrane invaginations. The OCS also serves as a fluid membrane source during the dramatic shape changes platelets undergo as they interact with the microenvironment. However, proteomics, lipidomics, and metabolomics are the primary tools for describing the platelet molecular status. When considering the dual immune/hemostatic phylogenetic origins of thrombocytes and platelets, one must note that their original role was to rapidly stop blood loss and eliminate circulating threats. Based on their rapid response to circulating tumor cells in cancer progression and metastasis, we have posited the notion of platelets as first responders—similar to their immediate influence in wounding, tissue repair, and inflammatory and immune responses [1–3]. Platelets play a critical role in threat mitigation, cancer biology, and metastasis and continue to be overlooked by commonly used sequencing technologies, which fail to detect all key biological changes in pathological samples.

## Platelets as first responders

Why first responders? This notion primarily stems from biophysical and functional characteristics coupled with a large numbers of platelets, compared to any other cell type, in circulation. Without an activating stimulus, platelets exist as small plate-like disks that maximize planar-surface interactions. The considerable number and biophysical characteristics of mammalian platelets make them more likely to be the first blood component to encounter and suppress the activity of tumor cells and virus particles. Again, due to their size and biophysical shape, platelets concentrate toward the outer laminar fluid-shear fields of flowing blood, enhancing encounters with vascular surfaces and the probability that their receptors will recognize foreign threats, vascular wall lesions, wounds, or tumors. In the case of a vascular-lesion basement membrane or underlying matrix exposure, platelets undergo receptor-mediated activation. This recognition process triggers (1) rapid breakdown of microtubular circular rings that support the plate-like structure, (2) further cytoskeletal actin polymerization, and (3) membrane changes to form filopodia, which lead to further adhesion, motility, and platelet recruitment to secure any gaps. This rapid process occurs within seconds coupled with exocytic release of storage granules that contain proteins, growth factors, cytokines, and lysozymes plus bioactive lipids, small molecules, and other host defenses (e.g., inflammation and hemostatic and wound factors). Short-term recruitment of additional platelets secures wounds, immobilizes foreign threats and immune cells, accelerates thrombogenesis, and initiates recruitment of fibroblasts and other immune cells. Unlike the thrombocytes that exist in lower vertebrates, mammalian platelets are rapidly expended during this process as no nucleus exists to support self-renewal. This formation of an unstructured, heterogenous, platelet-containing mass or clot is the rapid first response.

## Drones, drones everywhere!

Platelets functioning as expendable drones typically form in the bone marrow as membrane-bound organelle-containing cytoplasmic extrusions from the surfaces of megakaryocytes, the largest human multinucleate cell that grows larger with increasing ploidy (50–100 μm). Platelets, released into the blood stream from these megakaryocytes as small (2–3 μm) subcellular blebs [4], originate primarily in the bone marrow at an extremely high rate to achieve normal human-platelet concentrations of up to 400,000 per microliter. The estimated 10^11^ platelets produced each day are responsible for coagulation but can also impact viral and pathogen surveillance, cancer biology, and immune function. Pathogen uptake of small threats, such as virus particles, occurs in lower vertebrates which feature an extensive open-canalicular system retained in mammalian platelets whose numbers expanded exponentially as megakaryocytes developed to produce billions of platelets per day. Compared to lower vertebrate, 2-N thrombocytes, the mammalian megakaryocyte further improved upon the efficiency of platelet hemostasis compared to lower vertebrate, 2-N thrombocytes by dramatically increasing DNA content up to 128 N to significantly adapt to amplified bleeding during placentalization and immune responses. As a result, the mammalian megakaryocyte dramatically expanded platelet production in the protective bone-marrow microenvironment and the nucleus became expendable as platelets became more refined as circulating drones. Expanding on the drone theme, platelets also release a variety of vesicles upon activation to make up the bulk of blood-circulating microparticles and expand the available platelet surface area. Tumor-educated platelets appear to undergo modifications when they contact tumor cells, possibly retaining tumor-specific information including the primary tumor location [5].

## Speed is of the evolutionary essence

As thrombocytes evolved into platelets, they maintained a rapid-response ability when triggered by receptor-mediated activation and associated structural changes. Fully evolved platelets primarily function to mediate blood-loss-minimizing hemostasis when receptors recognize breaches in tissue integrity or vascular injury. This extremely rapid nature of platelet responses was linked to the evolution of prostaglandin metabolism, which in the yin-and-yang case of mammalian-platelet cyclooxygenase-1/thromboxane metabolism is balanced against cyclooxygenase-2/prostacyclin metabolism in the vascular endothelium. Both pathways engage highly labile, epoxide, metabolic intermediates with very short lived (~ 30 s) half-lives. The hemostatic aspect ensures that these very rapid and dramatic responses remain localized and do not become a breach in vascular integrity.

## To thrombose or not thrombose: that is the question

Along with a triggered coagulation cascade, platelets heighten the risk of pro-thrombotic events and contribute to cancer progression and metastasis. Cancer progression and the associated normal-tissue destruction function like persistent, chronic wounds. Cancers associated with increased thrombocytosis, platelet activation, platelet storage-granule release, and thrombosis may benefit from treatment with platelet agents to reduce cancer-metastasis risk. Contingent on individual patient risks, these factors act together to disrupt the tenuous balance between pro- and anti-coagulant processes, heightening thrombogenesis and possibly providing a biological niche for circulating-tumor cells to hide—thus promoting metastasis and cancer-cell survival following treatment and maintaining minimal residual disease (MRD).

## My lines, my lines, I forgot my lines

The merging of innate immunity and coagulation properties to confront injury and initiate repair is a phylogenetically ancient adaptation stemming from the early stages of eukaryotic and chordate evolution. Unfortunately, the lines of their scripted phylogenetic development become more difficult to follow as our focus on the fully evolved mammalian platelet increases in clinical venues. Invertebrate beginnings supported rapidly responding, large nucleated, amoeboid, granular hemocytes to secure an open circulatory system. The best example is the amebocyte-driven coagulation response to *Limulus* endotoxin as an archetypal function—ultimately handed down to human platelets—including the aggregation, adhesion, spreading, and storage-granule release of coagulation factors. The merging of this script remains apparent in nucleated thrombocytes of early chordates and the non-mammalian, egg-based vertebrates like fish, birds, amphibians, and reptiles that finally diverge due to a greater need to prevent the massive blood losses associated with placentalization and live birth. The lines of the mammalian-platelet immune function remain a largely unexplored part of the script, particularly in the case of tumor immunity.

## Overlooking the purloined platelet and its contents or why and how we tissue culture

Fetal-bovine calf serum provides a bulk source of pre-term, developmental, mammalian growth factors used to establish and grow human and other cell types in tissue culture [6]. Unstimulated platelets contain multiple storage granules: (1) alpha-granules (α-granules, proteins, cytokines growth factors); (2) dense granules (δ-granules, small molecules); (3) lysosomes (digestive and tissue remodeling enzymes); (4) microparticles (various platelet constituents); and (5) T-granules (toll-like receptors)—all released into clotted serum for use in tissue culture. Proteins released from α-granules include multiple C-X-C motif-(1,4,5,7,8 and 12) and C–C motif-(2,3,5,7 and 17) chemokines as well as growth and angiogenesis factors (e.g., platelet-derived growth factor, epidermal growth factor, transforming growth factor-β [TGFβ], and vascular endothelial growth factor). The small molecules released or synthesized include strongly bioactive, lipid prostaglandins (e.g., thromboxane A2) and lipoxygenase products (e.g., 12-hydroxyeicosatetraenoic acid) as well as serotonin and histamine. These highly reactive, small molecules remain as often-overlooked supplements when studying cell behavior in tissue culture if the serum is not stripped with activated charcoal to become fatty-acid free. Our dependency on bulk sources may have also limited our exploration of other more experimentally relevant sources of plasma. Have we missed opportunities to examine normal or cancer cell growth in matched patient plasma or normal controls? In one sense, then, the bulk of all modern-day tissue-culture-based biomedical science has been overshadowed and purloined by the fetal-calf platelet.

## Platelet exploration and uncharted immune functions

Platelet granule release is an essential immune-response component. During wound healing, platelets contribute to pathogen clearance, tissue repair, and the inflammation cycle. Receptor-mediated platelet responses help recognize and initiate clotting and release pro-inflammatory mediators that attract additional immune cells to sterilize the wound. As part of wound resolution, platelets release α2-macroglobulin, plasminogen activator, plasminogen, and plasminogen-activator inhibitor type-1 to help with fibrinolysis, clot dissolution, inflammation resolution, and wound repair. They also release tissue-remodeling enzymes, e.g., matrix metalloproteinases, involved in wound repair. Degranulation and platelet surface interactions with immune cells induce biological responses by leucocytes and progenitor and endothelial cells at the site of pathogen permeation or vascular injury inflow. Platelets interact with neutrophils, lymphocytes, and monocytes to activate and promote platelet leukocyte aggregates that immobilize and eliminate pathogens and tumor cells. Platelet toll-like receptors, among other immune recognition receptors, can engage pathogens and tumor cells leading to phagocytosis of smaller threats and identification of larger threats for elimination by other immune cells. Elevations in circulating platelet-to-lymphocyte ratios are associated with inflammation and poor outcomes and can be linked to infections, inflammatory diseases, and cancer. Mounting evidence supports the predictive value of platelet-to-lymphocyte ratios as a general biomarker of inflammation. Validation of this hypothesis will require a deeper analysis of platelet-lymphocyte interactions in prospective case–control studies.

## Trousseau’s legacy lives on

In 1865, Trousseau first reported migratory thrombophlebitis in cancer patients with elevated platelet counts or thrombocytosis. In ovarian cancer, paraneoplastic induction of thrombocytosis in orthotopic ovarian mouse models revealed tumor-derived, interleukin-6-stimulated, hepatic thrombopoietin synthesis which was supported by similar findings in patients [7]. Liver metastasis associated with *Fusobacterium* can also trigger the thrombotic Trousseau’s syndrome. Recent outcome data cancer prevention summaries have also highlighted the cancer-mitigating role of aspirin and other anti-platelet activation agents.

## Fast and furious! The gathering masses of platelet activation and aggregation and metastasis

Rapid platelet-based amplification responses help to stop bleeding and initiate fast and effective immune responses. High platelet counts often correlate with tumor invasiveness and metastasis and have a negative correlation with patient survival. Platelet hyperactivation, as a function of elevated platelet mean volume or aggregation, can be one predictor of cancer progression. During cancer progression and metastasis, platelet biochemical and biologic behavior is often modulated by tumor cells. Circulating tumor cells (CTCs) can initiate heterotypic tumor cell-induced platelet aggregation (TCIPA) [2, 8, 9]. Tumor cells can become entrapped within forming aggregates to become undetectable by other immune system components and protected from circulatory shear forces that lead to metastasis formation [10, 11]. Tumor cells also release thrombin which activates platelets and amplifies TCIPA. These thrombi may provide a supportive environment for CTCs by becoming tethered to blood vessels within distant tissues. Many studies have used anti-platelet drugs with chemotherapy to reduce platelet-mediated tumor cell survival and metastasis. Platelets can also promote angiogenesis by releasing angiogenic growth factors (e.g., vascular endothelial growth factor). Microfluidics can help isolate CTCs by targeting platelets that satellite on the tumor cell surfaces. A significant number of platelet-coated CTCs were found in patients with metastatic cancer with either epithelial- (lung or breast) or non-epithelial (melanoma) tumor origins or associations with leukocytes [12]. Isolating CTCs using platelet markers emphasize the potential of platelet-coated CTCs that go unnoticed by conventional isolation methods and their possible significance in metastasis.

## Dark side of the moon platelets in immune suppression

Like the dark side of the moon, we are only beginning to learn about the role of platelets in immune suppression. TGFβ activity enhances immunosuppression and provides a favorable environment for tumorigenesis. Platelets are the major source of latent TGFβ released during platelet activation. Elevated TGFβ levels initiate immunosuppression by blocking naive T-cell differentiation to type-1 T-helper effector phenotypes, promoting the conversion to Treg subset cells, and decreasing the antigen-presenting functions of dendritic cells. TGFβ also suppresses natural-killer cell activity and promotes the immunosuppressive M2 macrophage activity. Platelet activation also releases a furin-like proprotein convertase from platelets which in turn activates TGFβ. The activation and aggregation of platelets that elevate TGFβ and other growth factor release not only cause platelets binding with collagen but also provide a tumorigenic niche within these platelet-collagen traps to capture CTCs and promote their growth. The role of platelets in suppressing immune responses and the impact of platelet inhibition on the immune microenvironment require further study.

## Keep it small and simple platelets and minimal residual disease

Identifying potential tumors when they are small and simple is likely to help with treatment success. In this instance, minimal residual disease (MRD)—defined as disease remaining after curative-intent cancer therapy that predictably progresses into clinically detectable, locally recurrent, and/or distant metastases—is detected in patients with colorectal cancer by the highly sensitive, blood-based, prognostic biomarker circulating-tumor DNA (ctDNA). MRD detecting ctDNA assays allow oncologists to screen for cancer recurrence. If other platelet-based biomarkers (e.g., platelet-to-lymphocyte ratios or micrometastasis-educated platelets) can help to further identify ctDNA-defined MRD before clinically evident, macroscopic, the metastatic disease appears, this can improve the treatment window before tumor growth overtakes the immune system’s ability to eliminate the disease. Presently, little is known about how platelets impact the biology of micrometastases and whether anti-platelet treatments can enhance immunotherapy of MRD. Understanding the role of platelets in MRD will require expanding our discovery toolset beyond the commonplace protein and lipid nucleotide sequencing.

## In days of futures past and Waldo’s plight

The dual archetypal role of platelets in both coagulation and immune defense that arose from thrombocyte origins in days of futures past now requires more intensive study if we are to fully understand their biological function. The OCS found in thrombocytes and platelets provides information exchange and a fluid and responsive membrane source for the defense of the vascular microenvironment defense via recognition uptake of small and macromolecules and nanoscale particles such as viruses and tumor- or tissue-derived microvesicles. In this manner, platelets provide tumor- or disease-site education. This information is likely a static circulating snapshot of a tumor- or disease site since macromolecule recycling is not needed but would occur with nucleated thrombocytes. As well, a platelet-based information packet could possibly provide feedback to megakaryocyte lineage cell bone marrow to expand either platelet numbers or ploidy to produce billions of targeted platelets. With this information, informed or targeted platelets are likely to help decorate and immobilize threats, like CTCs or other pathogens that are too large to engulf. In the case of TCIPA and the immobilization of CTCs, opportunity exists for further and much more extensive interactions. We originally reported that extensive membrane interactions and interdigitation of cellular processes were on both the platelet- and tumor cell side of these heterotypic exchanges (see diagrammatic representation in Fig. 1) [3] The extent to which these result from platelets attempting to eliminate the CTC or the CTC defending itself remains unknown. This type of intravascular interaction may help explain the stochastic nature of metastasis depending on the ability of any given tumor cell to survive or use the platelet shield as a cloaking mechanism for immune defense. Alternately, the effectiveness of platelet cytokine release into the blood stream as part of its wounding or immune alert systems could also add to the stochastic nature of metastasis formation. Since these first responder reactions are immediate, many of these processes are likely to be conditionally governed by specific circumstances within the first microvascular bed that a tumor cell or other circulating threat encounters. Any increased formation of Trousseau’s syndrome-related thrombi coupled with increased interactions of platelets and tumor cells create a suitable environment for tumor cell entrapment. Given the complex, multi-faceted role of the platelet in coagulation, vascular defense, and cancer biology, it remains uncertain if cancer patients could benefit from not-yet-identified platelet-inhibition agents. As previously mentioned, evaluating these interactions morphologically is difficult since anucleate platelets rapidly become amorphous masses of cellular remnants once these reactions are triggered,

so where, indeed, Martin Handford, is Waldo in our multi-page biological illustration? As we routinely neglect to use the appropriate molecular methods of proteomics and the like to interrogate tumors and MRD in an RNA- or DNA-sequencing study of our cancer mural, is it time to revise how we search for this tiny, wooden-cane carrying, red- and white-striped beanie-clad subject in blue pants? If our minds remain unprepared or we fail to use the right tools, maybe Wally will never be found. For the sake of our pathogen-inflicted cancer patients, this particular biological-mural illustrator certainly hopes for better.

## Abbreviations

CTCs: Circulating tumor cells
MRD: Minimal residual disease
TCIPA: Tumor cell-induced platelet aggregation
TGFβ: Transforming growth factor-β
OCS: Open canalicular system

## References

[1] Kanikarla-Marie, P., Kopetz, S., Hawk, E. T., Millward, S. W., Sood, A. K., Gresele, P., et al. (2018). Bioactive lipid metabolism in platelet “first responder” and cancer biology. *Cancer and Metastasis Reviews,**37*(2–3), 439–454. 10.1007/s10555-018-9755-8 30112590

[2] Menter, D. G., Kopetz, S., Hawk, E., Sood, A. K., Loree, J. M., Gresele, P., et al. (2017). Platelet “first responders” in wound response, cancer, and metastasis. *Cancer and Metastasis Reviews,**36*(2), 199–213. 10.1007/s10555-017-9682-0 PMC5709140 28730545

[3] Menter, D., Davis, J., Tucker, S., Hawk, E., Crissman, J., Sood, A., et al. (2017). Platelets: “First responders” in cancer progression and metastasis. In P. Gresele, N. Kleiman, J. Lopez, & C. Page (Eds.), *Platelets in thrombotic and non-thrombotic disorders* (pp. 1111–1132). Springer Nature.

[4] Thon JN, Italiano JE (2010). Platelet formation. Seminars in Hematology.

[5] Best MG, Sol N, Kooi I, Tannous J, Westerman BA, Rustenburg F (2015). RNA-Seq of tumor-educated platelets enables blood-based pan-cancer, multiclass, and molecular pathway cancer diagnostics. Cancer Cell.

[6] Honn KV, Singley JA, Chavin W (1975). Fetal bovine serum: A multivariate standard. Proceedings of the Society for Experimental Biology and Medicine.

[7] Stone RL, Nick AM, McNeish IA, Balkwill F, Han HD, Bottsford-Miller J (2012). Paraneoplastic thrombocytosis in ovarian cancer. New England Journal of Medicine.

[8] Menter DG, Harkins C, Onoda J, Riorden W, Sloane BF, Taylor JD (1987). Inhibition of tumor cell induced platelet aggregation by prostacyclin and carbacyclin: An ultrastructural study. Invasion and Metastasis.

[9] Menter DG, Hatfield JS, Harkins C, Sloane BF, Taylor JD, Crissman JD (1987). Tumor cell-platelet interactions *in vitro* and their relationship to *in vivo* arrest of hematogenously circulating tumor cells. Clinical & Experimental Metastasis.

[10] Kanikarla-Marie P, Lam M, Menter DG, Kopetz S (2017). Platelets, circulating tumor cells, and the circulome. Cancer and Metastasis Reviews.

[11] Menter DG, Tucker SC, Kopetz S, Sood AK, Crissman JD, Honn KV (2014). Platelets and cancer: A casual or causal relationship: Revisited. Cancer and Metastasis Reviews.

[12] Jiang X, Wong KHK, Khankhel AH, Zeinali M, Reategui E, Phillips MJ (2017). Microfluidic isolation of platelet-covered circulating tumor cells. Lab on a Chip.

